# Efficacy of Marker-Based Motion Capture for Respiratory Cycle Measurement: A Comparison with Spirometry

**DOI:** 10.3390/s23249736

**Published:** 2023-12-10

**Authors:** Natalia D. Shamantseva, Tatiana A. Klishkovskaia, Sergey S. Ananyev, Andrey Y. Aksenov, Tatiana R. Moshonkina

**Affiliations:** 1 Pavlov Institute of Physiology, Russian Academy of Sciences, 6 Makarova Emb., Saint Petersburg 199034, Russia; ananevss@infran.ru (S.S.A.); moshonkina@infran.ru (T.R.M.); 2 Faculty of Information Measurement and Biotechnical Systems, Saint Petersburg Electrotechnical University “LETI”, 5 Professora Popova Str., Saint Petersburg 197022, Russia; tatianaklishkov@outlook.com (T.A.K.); a.aksenov@hotmail.com (A.Y.A.)

**Keywords:** respiratory rate, motion capture, optoelectronic plethysmography, spirometry, respiratory time components

## Abstract

Respiratory rate monitoring is fundamental in clinical settings, and the accuracy of measurement methods is critical. This study aimed to develop and validate methods for assessing respiratory rate and the duration leof respiratory cycle phases in different body positions using optoelectronic plethysmography (OEP) based on a motion capture video system. Two analysis methods, the summation method and the triangle method were developed. The study focused on determining the optimal number of markers while achieving accuracy in respiratory parameter measurements. The results showed that most analysis methods showed a difference of ≤0.5 breaths per minute, with R^2^ ≥ 0.94 (*p* < 0.001) compared to spirometry. The best OEP methods for respiratory rate were the abdominal triangles and the sum of abdominal markers in all body positions. The study explored inspiratory and expiratory durations. The research found that 5–9 markers were sufficient to accurately determine respiratory time components in all body positions, reducing the marker requirements compared to previous studies. This interchangeability of OEP methods with standard spirometry demonstrates the potential of non-invasive methods for the simultaneous assessment of body segment movements, center of pressure dynamics, and respiratory movements. Future research is required to improve the clinical applicability of these methods.

## 1. Introduction

In a clinical context, an irregular respiratory rate often indicates possible severe serious clinical outcomes [1,2]. Respiratory rates in healthy individuals can be influenced by various factors including physical activity, emotional states, and cognitive loads [3]. The importance of this monitoring extends beyond clinical scenarios into research and day-to-day life. Differentiating between phases of the respiratory cycle is crucial for certain medical interventions. For example, the effectiveness of nasal high-flow oxygen therapy is determined by the phase of respiration [4]. Understanding the expiratory and inspiratory phases is also essential when studying non-ventilatory behaviours, such as vocalisation, swallowing, or vomiting [5]. Managing the respiratory rate and distinguishing inhalation and exhalation phases, holds significance in evaluations related to postural stability [6,7].

Currently, over thirteen techniques are used to monitor the breathing rate (BR), and some are even remotely operated [8]. Contact-based techniques, such spirometry and inductive plethysmography, may reduce comfort due to the inconvenience of a face mask with a tube or sensors and cables. Non-contact methods like infrared thermography, radar and ultrasound offer advantages by allowing remote breathing detection and removing physical tethers. However, these methods require a complex setup and are often noise-sensitive, potentially affecting their accuracy [8]. Breathing patterns can be determined by analysing the movement of reflective markers in motion capture recordings. Optoelectronic plethysmography (OEP) utilises these markers placed on an individual’s torso to assess fluctuations in chest volume [9]. The accuracy of OEP directly correlates with the quantity of markers employed. However, both researchers and subjects of OEP assessments express a preference in reducing the number of markers to facilitate a more efficient preparation process. Highlighting the importance on marker count, Massaroni et al. demonstrated that 30-marker OEP configuration resulted in lower bias and limit of agreement (LOA) (bias = 0.056 L, LOA ± 0.35 L) compared to 89-marker OEP (bias = 0.16 L, LOA ± 0.4 L) [10]. Further research suggests that using fewer than 16 markers in OEP can still provide reliable estimates for respiratory rates and other breathing-associated parameters [11]. 

Postural variations play a significant role in influencing thoracoabdominal motion during quiet breathing. A study in healthy men showed that both craniocaudal and anteroposterior movements of the anterior surface of the pulmonary and abdominal rib cages were significantly greater in the sitting position than in the supine and right lateral positions [12]. A meta-analysis has been performed to evaluate the effect of body positioning on chest wall movement [13]. It was found that the sitting position improved the thoracic compartment of the chest wall, while the supine position resulted in superior improvement in the abdominal part compared to other body positions. Therefore, the accuracy of the non-contact monitoring methods that capture chest wall movements is likely to vary based on body position.

In our study, we used both OEP and spirometry to evaluate the breathing rate along with inspiratory and expiratory durations in sitting, standing and supine positions.

The main goal of this study was to develop and validate an OEP method to measure human external breathing to determine the BR and length of each phase in the respiratory cycle. We explored various methods to calculate respiratory data based on marker positions, with the aim of identifying the most accurate method for each of the three body positions. Another goal was to show that a minimum number of markers is sufficient to accurately determine selected respiratory parameters. 

The findings of this study may prove useful for understanding the relationship between posture and respiratory regulation. This method is also planned for use in subsequent research on contactless breathing rate recording, particularly in studies involving electrical spinal cord stimulation in patients with spinal cord injuries.

## 2. Materials and Methods

### 2.1. Subjects

Twenty-six healthy volunteers participated in the study (eleven males), with an age of 33 ± 11 years (range 18–55 years), and a ody mass index of 24.4 ± 2.8 kg/m^2^ (range 18.8–38.4 kg/m^2^). The exclusion criteria were any respiratory or musculoskeletal disorder, any symptoms of any kind of disease, medical/surgical procedure or trauma within four weeks of the initiation of the study, and pregnancy. On the day of the study, all volunteers assessed themselves as healthy. Post hoc analysis showed that the statistical power of the study was 94.6% based on the previously calculated BR in 368 patients in [14]. 

The 27th participant (female, 60 years, 18.9 kg/m^2^) was not included in the analysis due to the influence of wearing a tight shirt, which affected the quality of the data recording. 

The ethical approval for this study was granted by the ethics committee at the Pavlov Institute of Physiology of Russian Academy of Sciences (Minutes # 22-06 dated 3 November 2022). The study was conducted in strict adherence to the Declaration of Helsinki (World Medical Declaration of Helsinki, 2013). All participants were informed about the objectives and methodology of the study and signed the informed consent before participation.

### 2.2. Protocol

The pulmonary functional parameters of the participants were evaluated using spirography. The Tiffeneau–Pinelli index was assessed. This index is a calculated ratio obtained by dividing the forced expiratory volume in one second (FEV1) by the forced vital capacity (FVC) of the lungs. This index is instrumental in the precise diagnosis and continuous monitoring of various respiratory disorders [15].

Following this initial evaluation, respiratory indicators and OEP were recorded. Participants were guided into three different positions: sitting, standing, and supine. Each position was maintained for 100 s during the respiratory recordings. The participants were instructed to breathe naturally and maintain their usual breathing depth and rate.

### 2.3. Spirography and Spirometry

Pulmonary status was assessed by a clinical spirograph Diamant KM-AP-01 (Diamant LLC, Moscow, Russia).

For the recording of natural breathing in various body positions, a combined system, PowerLab C, Octal Bio Amp and a Spirometry Pod equipped with a 1000 L respiratory flow head (ADInstruments Pty Ltd., Bella Vista, Australia), was used. The system was set up to record the respiratory patterns in three distinct body positions.

Before starting the investigation, each participant’s breathing equipment was carefully calibrated using a one-liter calibration syringe.

### 2.4. Experimental Setup

A 10-camera motion capture system (Oqus 500+, Qualisys AB, Gothenburg, Sweden) was set up on the wall rails to record the 3D marker data at a frequency of 100 Hz. Participants were positioned in the center of a room that measured 6.4 × 5.5 m^2^ during data collection, where motion capture, spirometry, and OEP were used simultaneously. The Qualisys trigger helped synchronize the start of recording for both the motion capture system and the spirometer, ensuring that the data collected were aligned and accurate.

**Figure 1:**
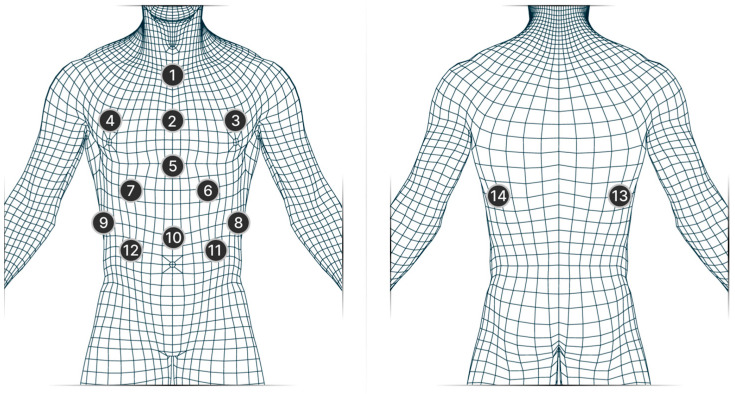
The placement of all markers used in the study; Table 1 gives a detailed description of each marker.

Fourteen passive markers were used to capture breathing patterns in both sitting and standing positions, as shown in Figure 1. However, only twelve markers were utilized when participants were in the supine position. Detailed description of the specific locations of each marker are shown in Table 1. Standard 12 mm Qualisys markers were placed on the participants’ bare skin and on a tightly fitted sports bra for females.

### 2.5. Methods for Calculating, Processing and Extracting Respiratory Curves

**Figure 2:**
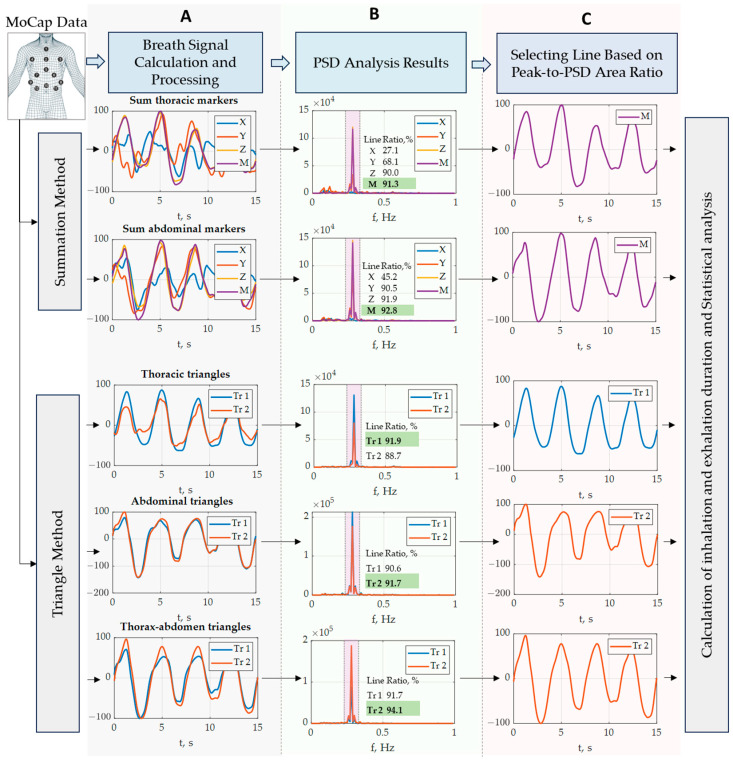
Methods for calculating, processing, and extracting respiratory curves were developed using optoelectronic plethysmography. (**A**): Filtering, smoothing, and normalization of the signal. (**B**): Spectrum constructed to show how the maximum peak area was selected for calculations, and table with peak area-to-total area ratios. (**C**): Extraction of respiratory curves using summation and triangle methods. MoCap: motion-captured. PSD: power spectral density.

Data were processed using MATLAB 2022b. Our objective was to calculate the respiratory curves based on data captured in various positions: standing, sitting, and supine. Two methods were implemented in MATLAB: the summation and triangle methods (Figure 2). These methods are based on the observation that the chest wall and abdomen move while breathing. 

#### 2.5.1. Summation Method

1. Markers were sorted into two categories based on their position to distinguish between thoracic and abdominal breathing regions:
  - Thoracic region (while in sitting and standing positions): SJN, M_Breast_1, L_Breast_1, R_Breast_1, M_Breast_2, R_Rib_2, L_Rib_2, R_Rib_1, and L_Rib_1.
  - Thoracic region (while in the supine position): SJN, M_Breast_1, L_Breast_1, R_Breast_1, M_Breast_2, R_Rib_1, and L_Rib_1.
  - Abdominal region (in all positions): R_Diaphragm, L_Diaphragm, R_Belly, L_Belly, and Belly_Center.
2. At each specific moment in time (*i*), calculations were made for the sum of marker projections onto the spatial axes (X*i*, Y_i_, Z_i_) as well as the sum of Euclidean distances from the markers to the origin of coordinates (M*_i_*).
  $$X_{i}=\sum_{j=1}^{n}x_{ij},Y_{i}=\sum_{j=1}^{n}y_{ij},Z_{i}=\sum_{j=1}^{n}z_{ij},M_{i}=\sum_{j=1}^{n}m_{ij}$$
  where j is the marker number from the region group.
3. The curve representing the sum of coordinate values was calculated. Four curves (X, Y, Z, M) were calculated for each of the thoracic and abdominal regions (Figure 2A). Each curve was subjected to additional processing and analysis to determine respiratory frequency.

#### 2.5.2. Triangle Method 

A pair of marker triangles were selected for each body region: thoracic, abdominal, and thoraco-abdominal. These triangles were used across all the body positions.

- Thoracic region. #1: SJN, M_Breast_1, L_Breast_1. #2: SJN, R_Rib_1, L_Rib_1.
- Abdominal region. #1: Belly_Center, L_Diaphragm, R_Diaphragm. #2: Belly_Center, L_Belly, R_Belly.
- Thoraco-abdominal region. #1: Belly_Center, R_Rib_1, L_Rib_1. #2: Belly_Center, R_Breast_1, L_Breast_1.

Empirical findings from multiple participants showed that marker M_Breast_1 in the first thoracic triangle showed better results than marker R_Breast_1. 

Calculation of the triangle’s area curve: Two curves were calculated for each region (Figure 2A). The Heron’s formula was used to calculate the area of the triangles.

$$S_{i}=\sqrt{p_{i}\left(p_{i}-a_{i}\right)\left(p_{i}-b_{i}\right)\left(p_{i}-c_{i}\right)}\;p_{i}\;=\;\frac{a_{i}+b_{i}+c_{i}}{2},$$

where $a_{i},\;b_{i},\;c_{i}$ are the lengths of the sides of the triangle, and $p_{i}$ is the semi-perimeter of the triangle at the *i*-th moment in time. 

Each curve was subjected to further processing and analysis to identify breathing frequency. 

#### 2.5.3. Data Processing

OEP data were filtered using a fourth-order zero-phase Butterworth high-pass filter with a cutoff frequency of 0.1 Hz to remove low-frequency components.

Then, signals were smoothed by applying a moving average filter with a span of 0.4 s and normalized to the peak value.

#### 2.5.4. Signal Selection

A power spectral density (PSD) analysis was performed for each signal. This analysis was used to identify the frequency with the maximum peak ($f_{max}$), which was associated with the breathing frequency (Figure 2B).

In each group, the area under the maximum PSD peak was computed for each signal, as shown in Figure 2B. For area calculation, the interval from $f_{max}-5$ counts to $f_{max}+5$ was used.

Subsequently, the ratio between the peak area and the total PSD area was calculated.

In each group, the signal with the maximum ratio was selected for further analysis. This resulted in the selection of one out of four curves for each region for the summation method, and one out of two curves for each region for the triangle method (Figure 2C).

#### 2.5.5. Curve Selection

Five distinct curves were chosen for comparison with the spirometry results. Two were from the summation method: the sum of the thoracic markers curve, and the sum of the abdominal markers curve. Three were from the triangle method: the abdominal triangle curve, thoracic triangle curve, and thoraco-abdominal triangles curve (Figure 2C).

### 2.6. Time Components Calculations

The inhalation and exhalation times were identified by detecting the extrema of all the respiratory curves (from spirometry and OEP).

Minima corresponded to the beginning of inhalation, and the maxima indicated the beginning of exhalation.

Key respiratory parameters, such as inspiratory time (T_ins_), expiratory time (T_exp_), and BR were determined.

### 2.7. Statistical Analysis

Statistical analysis of the breathing parameters was conducted using Statistica v.10.A software package. The goal was to evaluate the reliability and agreement between the OEP and spirometry data based on [16] and the EP09-A3 Guidelines for Measurement Procedure Comparison and Bias Estimation [17]. They suggest using plots of the difference to visualize the data, define the criteria for agreement in advance, and estimate the bias and limits of agreements (LOA) between two measurement procedures.

**Figure 3:**
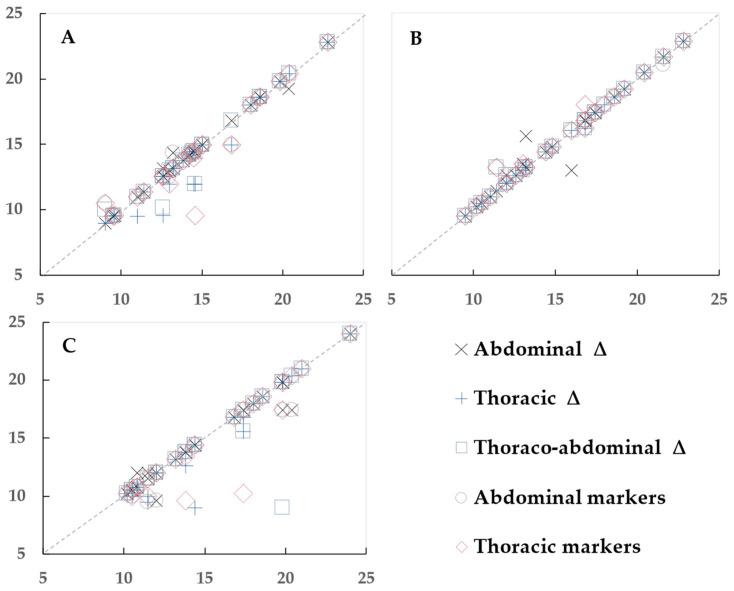
Scatter plots to visualize the relationship between breathing rate determined by spirometry (horizontal axis) and optoelectronic plethysmography (vertical axis), in breaths per minute. (**A**) Sitting, (**B**) standing, and (**C**) supine positions. Each data point represents one participant.

**Figure 4:**
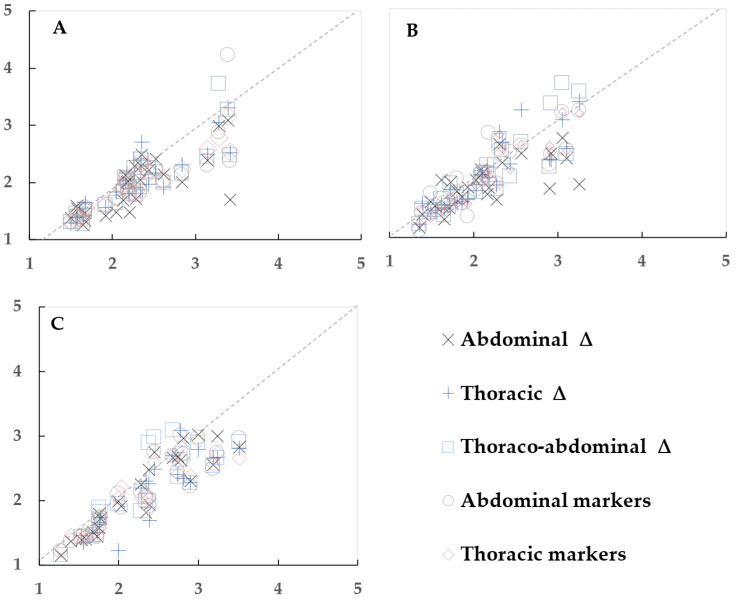
Scatter plots to visualize the relationship between inspiratory time determined by spirometry (horizontal axis) and optoelectronic plethysmography (vertical axis), in s. (**A**) Sitting, (**B**) standing, and (**C**) supine positions. Each data point represents one participant.

**Figure 5:**
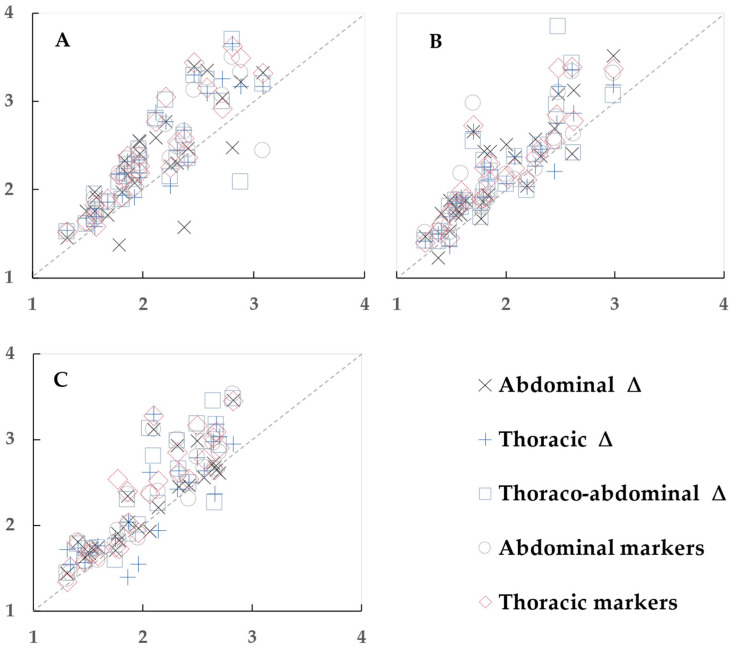
Scatter plots to visualize the relationship between expiratory time determined by spirometry (horizontal axis) and optoelectronic plethysmography (vertical axis), in s. (**A**) Sitting, (**B**) standing, and (**C**) supine positions. Each data point represents one participant.

1. The analysis began by plotting the differences to visualize the variation and trends between the OEP and spirometry data, with each data point representing one participant (Figure 3, Figure 4 and Figure 5). 

2. The Shapiro–Wilk criteria were used to assess the normality of the distribution of the respiratory parameters.

3. The mean and standard deviation were calculated for normally distributed data. Conversely, for non-normally distributed data, the median, along with the minimum and maximum range, were computed.

4. Estimation of bias and LOA between data obtained with spirometry and OEP was conducted using Bland–Altman analysis [18] in accordance with the recommendations of [16]. This step involved comparing the two measurement procedures to determine any systematic differences and their magnitudes.

5. Regression analysis was performed to establish the best-fitting model between spirometry and OEP data. The determination coefficient was calculated, and a *p*-value is given for methods that had a significant relationship (i.e., are interchangeable with spirometry).

6. The evaluation of the most efficient OEP method was performed in complex of the fourth and fifth steps of the analysis. Two criteria for the best agreement between OEP signal analysis and spirometry were the combination of minimum bias and maximum determination coefficient, with *p*-value < 0.05 [19].

Parameters that showed a non-normal distribution included the following:

- BR from spirometry in the supine position;
- BR from the thoracic triangles, thoraco-abdominal triangles, and the sum of the abdominal markers in the supine position;
- T_ins_ from thoraco-abdominal triangles in both sitting and standing positions;
- T_ins_ from the sum of abdominal markers in the sitting position.

## 3. Results

### 3.1. Participants 

The Tiffeneau–Pinelli indices of all participants, except participant #16, were within the normal range, indicating that they did not have respiratory symptoms [15]. Participant #16, a 36-year-old male, had a Tiffeneau–Pinelli index of 63%, indicating airway obstruction. Spirometry data of this subject were within three SDs of the averaged spirometry data of all other participants (Table S1). We analyzed his OEP data together with the data of the other participants.

A total of 78 recordings were taken with simultaneous recording of respiratory curves using spirometry and OEP. Four recordings in the supine position were excluded due to the position of the arms, which covered some of the markers. BR for one participant was not computed from thoracic markers in the supine position.

### 3.2. Breathing Rate

#### 3.2.1. Sitting Position

The mean spirometry BR in the sitting position was 14.6 ± 3.4 bpm.

All OEP methods showed good agreement with spirometry in the sitting position; however, two OEP methods (abdominal triangles and the sum of abdominal markers) showed the best agreement in two selected criteria (Table 2, Figure 3A). Bias between these methods and spirometry was <0.1% and R^2^ ≥ 0.97, *p* < 0.001. The average BR in abdominal triangles and the sum of abdominal markers was 14.6 ± 3.3 bpm, and best fit the spirometry value of BR.

#### 3.2.2. Standing Position

The spirometry BR in the standing position was 15.2 ± 3.7 bpm. 

All OEP methods showed good agreement with spirometry in the standing position. The best agreement between OEP and spirometry were with abdominal triangles, thoracic triangles, and the sum of abdominal markers (Table 3, Figure 3B). Bias between these methods and spirometry was ~0.1% and R^2^ ≥ 0.98, *p* < 0.001. Two other methods also showed R^2^ = 0.98, but the biases were greater. The best fit to the standing spirometry value of BR was BR achieved by abdominal and thoracic triangles (15.2 ± 3.7 bpm) and by the sum of abdominal markers (15.2 ± 3.6 bpm).

#### 3.2.3. Supine Position

The median spirometry BR in the supine position was 14.1 [10.2; 24] bpm. 

Two OEP methods (abdominal triangles and the sum of abdominal markers) showed the best agreement with spirometry (Table 4, Figure 3C). Bias between these methods and spirometry was less than 3% and R^2^ ≥ 0.94, *p* < 0.001. The thoracic triangles method showed R^2^ = 0.93, but the bias was greater. The BR of abdominal triangles (14.6 ± 3.8 bpm) and of the sum of abdominal markers (14.7 ± 4.1 bpm) were the best-fitted to the spirometry BR.

### 3.3. Inspiratory Time 

#### 3.3.1. Sitting Position

The spirometry T_ins_ in the sitting position was 2.30 ± 0.55 s. 

One OEP method, the sum of thoracic markers, showed best agreement with spirometry (Table 5, Figure 4A). The bias between the sum of thoracic triangles and spirometry was 13% and R^2^ = 0.87, *p* < 0.001. The T_ins_ of the sum of thoracic markers was 2.01 ± 0.46 s, which was in the best agreement with the spirometry T_ins_.

#### 3.3.2. Standing Position

The spirometry T_ins_ in the standing position was 2.1 ± 0.55 s. 

One OEP method, the sum of thoracic markers, showed the best agreement with spirometry (Table 6, Figure 4B). Bias was 12% and R^2^ = 0.85, *p* < 0.001. The T_ins_ of the sum of thoracic markers was 1.91 ± 0.49 s, which showed the best agreement with the spirometry T_ins_ in the standing position.

#### 3.3.3. Supine Position

The spirometry T_ins_ in the supine position was 2.26 ± 0.63 s. 

All but one of the OEP methods (thoraco-abdominal triangles) showed good agreement with spirometry (Table 7, Figure 4C). The best approximations of the supine T_ins_ determined by spirometry were from the T_ins_ of abdominal markers (2.1 ± 0.59 s), the sum of abdominal markers (2.08 ± 0.57 s), and the sum of thoracic markers (2.07 ± 0.53 s). Bias between these methods and spirometry was 8–9% and R^2^ ≥ 0.84, *p* < 0.001.

### 3.4. Expiratory Time

#### 3.4.1. Sitting Position 

The mean spirometry T_exp_ in the sitting position was 2.09 ± 0.46 s. 

Two OEP methods (thoracic triangles and the sum of thoracic markers) showed the best agreement with spirometry (Table 8, Figure 5A). Bias was 13–15% and R^2^ ≥ 0.81, *p* < 0.001. The best match to the spirometry T_exp_ determined in sitting position is T_exp_ by thoracic triangles (2.38 ± 0.60 s), and by the sum of thoracic markers (2.42 ± 0.62 s).

#### 3.4.2. Standing Position

The mean spirometry T_exp_ in the standing position was 1.94 ± 0.46 s. 

One OEP method, the sum of thoracic markers, showed the best agreement with spirometry (Table 9, Figure 5B). Bias was 14% and R^2^ = 0.801, *p* < 0.001. The T_exp_ by the sum of thoracic markers was 2.23 ± 0.57 s, and was the best fitted to spirometry T_exp_. 

#### 3.4.3. Supine Position

The mean spirometry T_exp_ in the supine position was 2.03 ± 0.48 s. 

Two OEP methods showed good agreement with spirometry; however, the best agreement was for the sum of abdominal markers (Table 10, Figure 5C). T_exp_ by the sum of abdominal markers was 2.28 ± 0.60 s. Bias was 11% and R^2^ = 0.82, *p* < 0.001.

## 4. Discussion

The aim of the study was to develop and validate methods using a motion capture system to determine respiratory rate and the duration of phases of the respiratory cycle in sitting, standing, and supine positions. We also aimed to determine the optimal number of markers for accurate measurement of respiratory parameters. 

We tested the usability of five sets of 5–9 spherical markers and two calculation methods to extract the respiratory signal. These methods are the summation method and the triangle method. For each body position, a signal with the maximum ratio of peak area (corresponding to respiratory frequency) to total PSD area was selected. The summation method divides the markers into thoracic and abdominal regions and calculates four different curves for each. The triangle method uses marker triangles for the three body regions and calculates area curves.

### 4.1. Accuracy of Monitoring Respiration Timing Components

**Figure 6:**
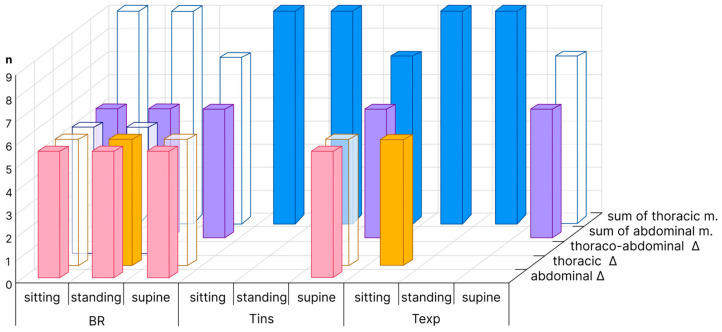
Selected best OEP analysis methods for BR, T_ins_ and T_exp_ in sitting, standing, and supine positions are solidly coloured. Methods with R^2^ > 0.80 are shown as transparent bars. n—is the number of markers required for measurement.

Two criteria were assessed for finding the best agreement between spirometry and OEP methods: a combination of the minimum bias and maximum determination coefficient (R^2^). Draper, using an applied regression analysis, suggests that the larger the R^2^, the better the fitted equation explains the variation in the data [19]. Chicco et al. concludes that R^2^ is more informative than mean absolute percentage error, and states that R^2^ > 0.80 indicates a very good regression model performance [20]. The number of acceptable OEP analysis methods is larger when this conclusion is considered. We selected the best OEP methods, and OEP methods with R^2^ > 0.80 are shown in Figure 6.

The guidelines for reporting reliability and agreement studies recommend focusing on the practical relevance of the results [16].

#### 4.1.1. Breathing Rate 

Measuring respiratory rates is fundamental in clinical settings [1,2]. In practice, observational BR monitoring is used routinely. In a single-day, multi-institutional, observational flesh-mob study with simultaneous data collection at six sites, researchers evaluated the accuracy of manual respiratory rate measurement in 368 hospitalised patients. It was found that the median automatically recorded BR was significantly higher (by 2 bpm, *p* < 0.001) than the median directly observed measurement [14]. Thus, the bias between observational and instrumental BR testing is routinely greater than 10%, and in practice, the demands on the accuracy of BR recording are not high.

We demonstrated that the OEP methods had a difference of less than 0.5 bpm (3%) for both the best agreement with spirometry and with R^2^ > 0.80 (Table 2, Table 3 and Table 4). Thus, most of the analysed methods are relevant for clinical and laboratory use.

The methods of abdominal triangles and of the sum of abdominal markers are the best in all body positions (Figure 6).

#### 4.1.2. Inspiratory Time. Expiratory Time

Although the measurement of BR has widespread clinical applications, we were unable to find any studies on the clinical significance and reference values for inspiratory and expiratory times. Normally, T_ins_ and T_exp_ are within a wide range in healthy subjects. Summarising the research in the literature that includes normal values for the respiratory time components [21], T_ins_ varies in the range of 1.5–2.2 s, and T_exp_ in the range of 2.1–2.9 s. All the values of these parameters that we measured fall within this range.

The measurement of T_ins_ is important to determine the fractional inspiratory time (FIT), i.e., T_ins_ × BF. FIT is ~0.45 for a normal breathing profile and less than 0.3 for an obstructed breathing profile [22,23]. We have shown that the best OEP methods for determining T_ins_ have a difference of ≤0.3 s (Table 5, Table 6 and Table 7). This bias results in an error of ~0.02 in the FIT calculation. Therefore, the best OEP analysis methods for T_ins_ are relevant for clinical use.

The sum of thoracic markers method is the best for T_ins_ recording in all tested body positions (Figure 6). The best OEP methods for determining T_exp_ also have a difference of ≤0.3 s (Table 8, Table 9 and Table 10). The sum of thoracic markers is the best for T_exp_ recording in the sitting and standing positions, and has an acceptable determination coefficient (>0.80) in the supine position (Figure 6). 

### 4.2. Marker Number Optimization

Previously, in the comparative study of four OEP methods, 89 markers were shown to be necessary for the measurement of temporal and volumetric respiratory parameters, but it was not concluded what the minimum number of markers would be for the measurement of respiratory rate and duration [10]. A recent study used twelve markers to compare the accuracy and reliability of three non-invasive devices for measuring respiratory rate in the supine position [24].

The feasibility of using a reduced number of markers to obtain reliable data was also investigated in our study. For the triangle method, we calculated two sets of triangles for each of the three regions (abdominal, thoracic, and thoraco-abdominal). It was not possible to determine only one set of triangles for each of the body positions. Therefore, the set of two triangles is required for the algorithm to further calculate the most optimal (based on maximum ratio) set for each position. The use of all triangles methods as well as the sum of abdominal markers in all positions requires five markers, whereas the summation of thoracic markers in the supine position requires seven markers and nine markers in the sitting and standing positions.

Five markers provide an accurate measurement of BR in all body positions using either the abdominal triangle method or the sum of the abdominal markers (Figure 6). This number of markers is sufficient to record T_ins_ in the supine position and T_exp_ in the supine and sitting positions. To determine T_ins_ in the sitting and standing positions and T_exp_ in the standing position, nine markers are required. If a study aims to determine all respiratory time components in sitting, standing and supine positions, 7–9 markers are required (Figure 6).

Thus, we have reduced the number of markers necessary and sufficient to accurately record BR compared to known studies. We have shown that 5–9 markers are sufficient to determine T_ins_ and T_exp_.

### 4.3. Potential Application—Study of Posture-Respiratory Relationship

Fundamental studies show that there is a direct relationship between respiratory and postural control systems [25,26]. In healthy adults, movement of the hips and lower extremities actively attempts to compensate for respiratory-induced postural perturbances through small angular shifts to maintain stability [26]. Body position has also been shown to influence chest wall motion [12,13]. Non-contact methods of breathing control are required to simultaneously assess the movement of the body segments, center of pressure dynamics, and respiratory movements. Standard spirometry cannot be used for such tasks due to its direct influence on posture in standing and sitting positions. Inductive plethysmography slightly affects the movement of the chest wall during breathing.

Measuring the relationship between postural control and respiration has clinical relevance in older adults [27] and in the assessment of cortical structures (motor, premotor, and supplementary motor areas) [28]. 

## 5. Limitations and Future Directions 

Several limitations should be considered. First, there is an absence of clinical criteria for selecting bias for T_ins_ and T_exp_, and the results are currently applicable primarily to healthy young individuals. Additionally, OEP methods may not be suitable for sleep monitoring or long-term intensive care unit patient monitoring. Furthermore, measurements must be taken without upper clothing. 

Future directions for research include the synchronization of video data with OEP and spirometry, improving camera resolution and placement, and accounting for periods of restless breathing, such as sneezing, speaking, swallowing, and coughing, in clinical research. These advancements could enhance the clinical applicability and robustness of respiratory monitoring methods. This non-invasive method shows promise for the simultaneous monitoring of postural balance and external breathing patterns in adults.

## 6. Conclusions

The present study establishes the interchangeability of OEP methods with standard spirometry for monitoring respiratory time components in sitting, standing and supine positions. OEP-based BR showed a bias of less than 0.5 breaths per minute (3%) compared to standard spirometry in all registered positions. For T_ins_ and T_exp_, the best OEP methods showed a difference of less than or equal to 0.3 s compared to spirometry. To our knowledge, the study appears to be the first to measure inspiratory and expiratory times in three different positions using a non-contact method. We have shown that 5–9 markers are sufficient to accurately determine the respiratory time components in the sitting, standing, and supine positions. The results of the study will be used to further investigate the relationship between postural control and respiration.

## Figures and Tables

**Table 1:** Marker location. Marker numbers are the same as those shown in Figure 1.

Marker Number	Marker Name	Marker Location
1	SJN	the interclavicular fossa
2	M_Breast_1	body of the sternum
3	L_Breast_1	left of the mid-clavicular line at the level of the fourth rib
4	R_Breast_1	Right of the mid-clavicular line at the level of the fourth rib
5	M_Breast_2	xiphoid process
6	L_Diaphragm	left of the anterior surface of the chest at the level of the ninth rib
7	R_Diaphragm	right of the anterior surface of the chest at the level of the ninth rib
8	L_Rib_1	left of the lateral surface of the chest at the level of the tenth rib
9	R_Rib_1	right of the lateral surface of the chest at the level of the tenth rib
10	Belly_center	above the navel
11	L_Belly	left of the mid-clavicular line at the navel level
12	R_Belly	right of the mid-clavicular line at the navel level
13	R_Rib_2	Right of the middle of the spine of the scapula at the level of the tenth rib
14	L_Rib_2	left of the middle of the spine of the scapula at the level of the tenth rib

**Table 2:** Selection of the most efficient OEP methods for seated breathing rate. Average ± SD, in bpm. Bias ± LOA, in bpm. R^2^: determination coefficient. Best agreement between spirometry and OEP is in bold. * *p* < 0.001.

	Triangle Method			Summation Method	
	Abdominal Triangles	Thoracic Triangles	Thoraco-Abdominal Triangles	Abdominal Markers	Thoracic Markers
Average ± SD	**14.6 ± 3.3**	14.1 ± 3.7	14.4 ±3.5	**14.6 ± 3.3**	14.3 ±3.5
Bias ± LOA	**0.01 ± 0.70**	−0.47 ± 1.80	−0.15 ± 1.40	**0.01 ± 1.05**	−0.26 ± 2.30
R^2^	0.99 *	0.93 *	0.95 *	0.97 *	0.90 *

**Table 3:** Selection of the most efficient OEP methods for standing breathing rate. Average ± SD, in bpm. Bias ± LOA, in bpm. R^2^: determination coefficient. The best agreement between spirometry and OEP is in bold. * *p* < 0.001.

	Triangle Method			Summation Method	
	Abdominal Triangles	Thoracic Triangles	Thoraco-Abdominal Triangles	Abdominal Markers	Thoracic Markers
Average ± SD	**15.2 ± 3.7**	**15.2 ± 3.7**	15.3 ± 3.6	**15.2 ± 3.6**	15.3 ± 3.6
Bias ± LOA	**0.02 ± 1.56**	**−0.02 ± 0.23**	0.06 ± 0.76	**0.02 ± 0.77**	0.11 ± 0.87
R^2^	0.98 *	0.99 *	0.98 *	0.98 *	0.98 *

**Table 4:** Selection of the most efficient OEP methods for supine breathing rate. Average ± SD or median [minimum, maximum], in bpm. Bias ± LOA, in bpm. R^2^: determination coefficient. The best agreement between spirometry and OEP is in bold. * *p* < 0.001.

	Triangle Method			Summation Method	
	Abdominal Triangles	Thoracic Triangles	Thoraco-Abdominal Triangles	Abdominal Markers	Thoracic Markers
Median [min, max] or average ± SD	**14.6 ± 3.8**	13.5 [9.6; 24.0]	13.8 [9.0; 24.0]	**14.7 ± 4.1**	13.3 [9.6; 24.0]
Bias ± LOA	**−0.24 ± 1.82**	−0.52 ± 2.33	−0.52 ± 4.34	**−0.39 ± 1.78**	−0.62 ± 3.22
R^2^	0.94 *	0.93 *	0.74 *	0.95 *	0.85 *

**Table 5:** Selection of the most efficient OEP method for seated T_ins_. Average ± SD or median [minimum, maximum], in s. Bias ± LOA, in s. R^2^: determination coefficient. The best agreement between spirometry and OEP is in bold. * *p* < 0.001.

	Triangle Method			Summation Method	
	Abdominal Triangles	Thoracic Triangles	Thoraco-Abdominal Triangles	Abdominal Markers	Thoracic Markers
Median [min, max] or average ± SD	1.94 ± 0.49	2.03 ± 0.50	1.97 [1.28; 3.73]	1.94 [1.31; 4.23]	**2.01 ± 0.46**
Bias ± LOA	−0.37 ± 0.64	−0.28 ± 0.47	−0.27 ± 0.44	−0.30 ± 0.48	**−0.30 ± 0.50**
R^2^	0.55 *	0.78 *	0.76 *	0.69 *	0.87 *

**Table 6:** Selection of the most efficient OEP method for standing T_ins_. Average ± SD or median [minimum, maximum], in s. Bias ± LOA, in s. R^2^: determination coefficient. The best agreement between spirometry and OEP is in bold. * *p* < 0.001.

	Triangle Method			Summation Method	
	Abdominal Triangles	Thoracic Triangles	Thoraco-Abdominal Triangles	Abdominal Markers	Thoracic Markers
Median [min, max] or average ± SD	1.82 ± 0.35	1.95 ± 0.51	1.87 [1.21; 3.36]	1.95 ± 0.48	**1.91 ± 0.49**
Bias ± LOA	−0.32 ± 0.61	−0.21 ± 0.53	−0.19 ± 0.45	−0.20 ± 0.65	**−0.25 ± 0.51**
R^2^	0.48 *	0.75 *	0.78 *	0.74 *	0.85 *

**Table 7:** Selection of the most efficient OEP methods for supine T_ins_. Average ± SD, in s. Bias ± LOA, in s. R^2^: determination coefficient. The best agreement between spirometry and OEP is in bold. * *p* < 0.001.

	Triangle Method			Summation Method	
	Abdominal Triangles	Thoracic Triangles	Thoraco-Abdominal Triangles	Abdominal Markers	Thoracic Markers
Average ± SD	**2.1 ± 0.59**	2.07 ± 0.60	2.21 ± 0.58	**2.08 ± 0.57**	**2.07 ± 0.53**
Bias ± LOA	**−0.18 ± 0.73**	−0.21 ± 1.06	−0.16 ± 0.82	**−0.18 ± 0.67**	**−0.20 ± 0.59**
R^2^	0.84 *	0.803 *	0.70 *	0.85 *	0.85 *

**Table 8:** Selection of the most efficient OEP methods for seated T_exp_. Average ± SD, in s. Bias ± LOA, in s. R^2^: determination coefficient. The best agreement between spirometry and OEP is in bold. * *p* < 0.001.

	Triangle Method			Summation Method	
	Abdominal Triangles	Thoracic Triangles	Thoraco-Abdominal Triangles	Abdominal Markers	Thoracic Markers
Average ± SD	2.33 ± 0.59	**2.38 ± 0.60**	2.37 ± 0.57	2.39 ± 0.54	**2.42 ± 0.62**
Bias ± LOA	0.24 ± 0.68	**0.28 ± 0.46**	0.25 ± 0.44	0.29 ± 0.50	**0.31 ± 0.54**
R^2^	0.61 *	0.81 *	0.63 *	0.74 *	0.83 *

**Table 9:** Selection of the most efficient OEP method for standing T_exp_. Average ± SD, in s. Bias ± LOA, in s. R^2^: determination coefficient. The best agreement between spirometry and OEP is in bold. * *p* < 0.001.

	Triangle Method			Summation Method	
	Abdominal Triangles	Thoracic Triangles	Thoraco-Abdominal Triangles	Abdominal Markers	Thoracic Markers
Average ± SD	2.18 ± 0.56	2.18 ± 0.55	2.20 ± 0.61	2.18 ± 0.56	**2.23 ± 0.57**
Bias ± LOA	0.24 ± 0.61	0.23 ± 0.50	0.24 ± 0.41	0.24 ± 0.55	**0.27 ± 0.49**
R^2^	0.76 *	0.76 *	0.70 *	0.72 *	0.801 *

**Table 10:** Selection of the most efficient OEP method for supine T_exp_. Average ± SD, in s. Bias ± LOA, in s. R^2^: determination coefficient. The best agreement between spirometry and OEP is in bold. * *p* < 0.001.

	Triangle Method			Summation Method	
	Abdominal Triangles	Thoracic Triangles	Thoraco-Abdominal Triangles	Abdominal Markers	Thoracic Markers
Average ± SD	2.26 ± 0.54	2.26 ± 0.60	2.44 ± 0.66	**2.28 ± 0.60**	2.33 ± 0.62
Bias ± LOA	0.20 ± 0.67	0.28 ± 1.02	0.31 ± 0.68	**0.23 ± 0.57**	0.29 ± 0.54
R^2^	0.75 *	0.67 *	0.74 *	0.82 *	0.801 *

## Data Availability

The datasets generated and/or analyzed during the current study are available from the corresponding author upon reasonable request.

## Supplementary Materials

The following supporting information can be downloaded at: https://www.mdpi.com/article/10.3390/s23249736/s1, Table S1: Averaged spirometry BR, T_ins_ and T_exp_ of 25 participants (without one) and one participant (#16) with reduced Tiffno index, in s.
