# Supplementary material for: Efficacy of Marker-Based Motion Capture for Respiratory Cycle Measurement: A Comparison with Spirometry
Source: Sensors (Basel). 2023 Dec 10;23(24):9736. doi: 10.3390/s23249736 (PMC10748239; doi:10.3390/s23249736)
Supplement: Supplementary file 1 [file sensors-23-09736-s001.zip › sensors-2725864-supplementary.pdf]

Table S1. Averaged spirometry BR, T_{ins} and T_{exp} of 25 participants (without one) and one participant (#16) with reduced Tiffno index, in sec.

№	Sitting			Standing			Supine		
	BR	T_{ins}	T_{exp}	BR	T_{ins}	T_{exp}	BR	T_{ins}	T_{exp}
N = 25	14.6 ± 3.4	2.27 ± 0.55	2.07 ± 0.46	15.3 ± 3.6	2.12 ± 0.55	1.93 ± 0.46	15.3 ± 4.1	2.26 ± 0.63	2.01 ± 0.48
#16	11.4	2.83	2.57	12.6	2.43	2.46	10.2	2.98	2.72
